# Bringing everyone to the table – findings from the 2018 Phelan-McDermid Syndrome Foundation International Conference

**DOI:** 10.1186/s13023-020-01389-6

**Published:** 2020-06-16

**Authors:** Kimberly Goodspeed, Geraldine Bliss, Diane Linnehan

**Affiliations:** 1 Department of Pediatrics, Neurology & Neurotherapeutics, and Psychiatry, University of Texas Southwestern Medical Center, 5323 Harry Hines Blvd, Dallas, TX 75390 USA; 2 Phelan-McDermid Syndrome Foundation, P.O. Box 1153, 8 Sorrento Drive, Osprey, FL 34229 USA

**Keywords:** Phelan-McDermid syndrome, Autism Spectrum disorder, SHANK3, 22q13, Patient Foundation, Patient involvement

## Abstract

**Background:**

Phelan-McDermid Syndrome (PMS) is a rare neurodevelopmental disorder characterized by global developmental delay, autism spectrum disorder, and numerous systemic complications including seizures, gastrointestinal dysfunction, and renal anomalies. The Phelan-McDermid Syndrome Foundation (PMSF) was created to improve the quality of life of people affected by PMS worldwide by supporting families, accelerating research, and raising awareness. To further this mission, the PMSF initiated the Phelan-McPosium in 2016 to bring families affected by PMS, clinicians, and researchers together to design patient-centered rigorous clinical and translational research. Here, we present findings from the 2018 Phelan-McPosium.

**Results:**

The 2018 Phelan-McPosium was attended by 183 families and 35 researchers and clinicians. Overall, the Early Childhood parents raised the fewest number of concerns, families of Late-Childhood patients raised more concerns around epilepsy and behavioral problems, and Teen and Adult families were primarily concerned about implications of genetic testing, gastrointestinal dysfunction, and regression. All families were concerned with feasibility, safety and importance of clinical trials for PMS.

**Conclusions:**

The concerns raised by families across the sessions varied by age in a manner which may overlap with the emergence of various signs and symptoms through the natural history of PMS. The design of the Phelan-McPosium session has successfully generated thoughtful research questions that led to innovative investigations and clinical trials that are shaping the standard of care for PMS. This is an approach which could be employed by any rare disease group to align translational research efforts with a patient-centered focus.

## Background

Phelan McDermid Syndrome (PMS; OMIM # 606232) is a rare neurogenetic syndrome with a broad phenotypic spectrum. Most individuals present with developmental delay with severely impaired language (> 75%), hypotonia (75%), and dysmorphic features [1]. Autistic traits (84%), behavioral problems such as aggression (28%), and seizures (27%) are commonly reported, and systemic involvement including gastrointestinal dysfunction (constipation 41%, gastroesophageal reflux 42%), renal anomalies (26%), and disrupted sleep (46%) are prevalent [1, 2]. The majority of published clinical data is in the form of case reports and case series, which have been reviewed in detail by Kolevzon et al. [3]. It is well-accepted that PMS is caused by deletions or mutations leading to a haploinsufficiency of *SHANK3*, found on chromosome 22q13.3, though a substantial number of patients carry a large deletion involving numerous genes in addition to *SHANK3* [3–5]. Approximately 75% of individuals with PMS carry a terminal deletion of 22q13.3 [6]. It is believed to be de novo in most cases, though a balanced translocation is found in 20% of parents of PMS patients, and cases of parental germline mosaicism have been suspected in cases in which siblings carry an identical variant [3]. The mainstay of treatment available for PMS is supportive care and management of comorbidities to target behavioral problems, sleep problems, seizures, and gastrointestinal issues as outlined in the practice parameters published in 2014 [3]. The Phelan McDermid Syndrome Foundation (PMSF) has been instrumental in organizing research efforts across disciplines and funding endeavors that have the potential to transform the way PMS is treated.

PMSF prides itself in being the global leader in providing support for those affected by Phelan-McDermid Syndrome. The foundation was created by parents and researchers and has maintained a culture of global inclusion and collaboration throughout its history. PMSF is engaged in agreements with global partners as well as a team of regional representatives. Global partners are international, legally recognized organizations that provide support to families of individuals with Phelan-McDermid Syndrome, while regional representatives include parent-to-parent volunteers who support and inform families in their region about research, family support, and advocacy efforts of PMSF. The PMSF created the Phelan-McPosium to be a structured session within the larger family conference in 2016 as a way to share the most recent findings from clinical research and to give families a voice in the design and conduct of future research studies. The overarching mission of the Phelan-McPosium is to improve patient-centered outcomes research. The immediate aims of the meeting are 1) to collect first-hand information from families about their experiences, questions, and priorities, and 2) to return lay-friendly, current research findings back to families. The long-term goals are 1) to shape the development of new research studies which reflect the greatest concerns and priorities of families and lead to the development of new treatments, therapies, or clinical guidelines for people with PMS and 2) to help families make the best decisions for their children. As a result of the 2016 Phelan-McPosium discussions among families and researchers, the ongoing Natural History Study was expanded to include adult PMS patients and a new microbiome study was launched. The 2018 PMSF International Family Conference was planned entirely by a team of family members and PMSF staff and was held in Dallas, Texas. The McPosium took place over two half-days of the conference. While the remainder of the conference includes time for family sessions and detailed informational sessions on PMS research and clinic topics of interest, the McPosium structure brings advocates “to the table” with researchers, enabling direct communication about the issues that most significantly impact the patient’s quality of life. Here we review the findings from 2018 McPosium.

## Results

### The PMSF is comprised of 974 US members and 1054 international members

In total, the PMSF is comprised of 974 US members and 1054 international members. The 2018 Phelan-McPosium was attended by 183 families and 35 researchers traveling from the US and 11 countries abroad including Australia, Brazil, Canada, France, Great Britain, Ireland, Malaysia, Norway, Spain, South Africa, and Venezuela (Table 1). Thirteen patient representatives (including author GB) from global partner organizations were in attendance at the conference and Phelan-McPosium in 2018. Experts from both domestic and international sites presented their work at the Phelan-McPosium, alongside parent volunteers (Table 2). PMSF provided financial assistance to four international families (Spain, France, Malaysia, and Ireland) to cover the cost of registration and accommodations. The regional representatives (including author DL) met at the conference to share topics of interest with the group. PMSF continues to improve the infrastructure of the organization in order to support as many families as possible, filling the communication gap amongst all stakeholders.

**Table 1:** Regional membership in the Phelan-McDermid Syndrome Foundation and 2018 PMSF International Conference attendance. The majority of attendees were from regions within the US, but an additional eight international representative were also in attendance

**US Regions**	N (%)	Conference Attendance 2018
REGION 1 NORTH: AK, ID, OR, WA, WY	45 (5)	
REGION 1 SOUTH: CA, HI, NV	112 (11)	
REGION 2: IA, MN, NE, ND, SD, WI	73 (7)	
REGION 3 NORTH: KS, AR, MO, OK	61 (6)	
REGION 3 SOUTH: LA, TX	85 (9)	
REGION 3 WEST: UT, AZ, CO, NM	42 (4)	
REGION 4 CENTRAL: PA, NY, NJ	122 (13)	
REGION 4 NORTH: VT, NH, ME, RI, CT, MA	45 (5)	
REGION 4 SOUTH: DE, MD, WV, VA, WASHINGTON DC	61 (6)	
REGION 5 NORTH: TN, NC, SC	56 (6)	
REGION 5 SOUTH: AL, FL, GA, MS	99 (10)	
REGION 9: IN, IL, KY, MI, OH	171 (18)	
US Total (including 2 unreported State)	974	158
**International Regions**	N (%)	
REGION 6: Ireland & UK	252 (24)	2
REGION 7 WEST: Alberta, British Columbia, Manitoba, Saskatchewan	38 (4)	14
REGION 7 EAST: New Brunswick, Newfoundland, Nova Scotia, Ontario, Prince Edward Island, Quebec	58 (6)	
REGION 8: Australia, New Caledonia, New Zealand, India	130 (12)	4
REGION 10: Mexico	7 (1)	
REGION 11: Central America	3 (< 1)	
REGION 11: Brazil	85 (8)	2
REGION 12 NORTH: Spain	66 (6)	1
REGION 12 SOUTH: Portugal	8 (1)	
REGION 13: Belgium, France, Luxembourg	112 (11)	1
REGION 14: Germany, The Netherlands	59 (6)	
REGION 15: Italy	36 (3)	
REGION 16: Denmark, Finland, Norway, Sweden	47 (4)	1
REGION 17: Albania, Austria, Bosnia, Bulgaria, Croatia, Czech Republic, Hercegovina, Hungary, Macedonia, Moldova, Romania, Slovenia, Switzerland	31 (3)	
REGION 18: Brunei, Indonesia, Malaysia, Philippines, Singapore, Thailand	22 (2)	1
REGION 19: Poland	12 (1)	
REGION 20: China, Taiwan	17 (2)	
REGION 21: Greece, Turkey, Israel	27 (3)	
REGION 22: Russian Federation	3 (< 1)	
REGION 23: South Africa	1	1
Total (including 18 unreported Country)	1032	185

**Table 2:** PMS experts participating in the 2018 PMSF International Family Conference summarizing their respective roles in the conference as well as current practice location

PMS Expert	Location	Participation
Alex Kolevzon, MD	Clinical Director Seaver Autism Center Icahn School of Medicine at Mount Sinai New York, New York, United States	Lecture: Clinical trials Panel: Challenging behaviors & Clinical trials
Ann Neumeyer, MD	Massachusetts General Hospital for Children Clinical Director of the Lurie Center for Autism Harvard Medical School Lexington, Massachusetts, United States	Panel: Regression
Audrey Thurm, PhD	National Institutes of Health Bethesda, Maryland, United States	Panel: Challenging behaviors
Catalina Betancur, MD, PhD	INSERM Paris, France	Panel: Genetics
Dean Hartley, PhD	Autism Speaks New York, New York, United States	Panel Moderator: Genetics
Eva Loth, PhD	EU-AIMS SFARI Investigator Kings College London London, United Kingdom	Panel: Challenging behaviors
Jimmy Holder, MD, PhD	Texas Children’s Hospital Baylor College of Medicine Houston, Texas, United States	Lecture: Epilepsy Panel: Epilepsy
Joe Buxbaum, PhD	Seaver Autism Center Icahn School of Medicine at Mount Sinai New York, New York, United States	Lecture: Clnical trials Panel: Clinical trials
Joe Horrigan, MD	AMO Pharma Wayne, Pennsylvania, United States	Panel: Epilepsy
Joe Bernstein, MD, PhD	Lucile Salter Packard Children’s Hospital Stanford Medicine Stanford, California, United States	Lecture: Regression Panel: Regression & Genetics
Katy Phelan, PhD	Florida Cancer Specialists Fort Myers, Florida, United States	Panel: Genetics
Kent Williams, MD	Nationwide Children’s The Ohio State University College of Medicine Columbus, Ohio, United States	Lecture: GI Panel: GI
Latha Soorya, PhD	Rush Medical College Chicago, Illinois, United States	Panel: Challenging behaviors
Liz Berry-Kravis, MD, PhD	Rush Medical College Chicago, Illinois, United States	Panel: Clinical trials & challenging behaviors
Nathan Call, PhD, BCBA-D	Marcus Autism Center Emory University School of Medicine Atlanta, Georgia, United States	Lecture: Challenging behaviors Panel: Challenging behaviors
Pilar Magoulas, MS, CGC	Texas Children’s Hospital Baylor College of Medicine Houston, Texas, United States	Lecture: Genetics Panel: Genetics
Ruth Ann Luna, PhD	Texas Children’s Hospital Baylor College of Medicine Houston, Texas, United States	Panel: GI
Siddharth Srivastava, MD	Boston Children’s Hospital Harvard Medical School Boston, Massachusetts, United States	Panel: Epilepsy
Tesi Kohlenberg, MD	PMS Parent, MD Child and Adolescent Psychiatrist	Panel Moderator: Regression
Yong-Hui Jiang, MD, PhD	Duke University School of Medicine Durham, North Carolina, United States	Panel: Genetics
Chris Winrow, PhD	PMS Parent Ironwood Pharmaceuticals	Panel Moderator: Sleep
Rob Ring, PhD	Autos Consulting & Advisory Solutions Philadelphia, Pennsylvania, United States	Panel Moderator: Challenging behaviors
Bill Bennett, MD, MS	Indiana University School of Medicine PMS Parent Indianapolis, Indiana, United States	Panel Moderator: GI
Abby Lievense Lagunoff	PMS Parent Co-Organizer	
Geraldine Bliss	PMS Parent Co-Organizer	Panel moderator: Epilepsy
Craig Powell, MD, PhD	Civitan International Research Center University of Alabama Birmingham Birmingham, Alabama, United States	Concluding remarks
Julie Wess	PMS Parent	PollEverywhere Coordinator

### Session 1: Clinical Trials Update, Joe Buxbaum, PhD, Icahn School of Medicine at Mount Sinai

This session reviewed the basics of translational science, provided preliminary and published results from recent clinical trials, and updated attendees on future directions. PMS, together with Tuberous Sclerosis Complex and PTEN Hamartoma Tumor Syndrome, form the Rare Disease Clinical Research Network Developmental Synaptopathies Consortium. Through the support of this consortium, the PMSF has executed natural history studies that have identified biomarkers and laid the foundation to test novel therapeutics in multisite clinical trials. Based on PMS animal model research that demonstrated immature neuronal synaptic development, insulin-like growth factor (IGF-1), a compound that supports synaptic maturation, emerged as a possible treatment. IGF-1 is currently FDA-approved for use in children with growth hormone deficiencies. It has demonstrated improvements in brain function in animal models treated with IGF-1 and improvements in repetitive behaviors in a small pilot study of PMS patients [7–9]. The next step is to conduct a larger clinical trial to investigate the safety and tolerability of IGF-1 in PMS patients as well as assess efficacy using a battery of neurobehavioral scales. Dr. Buxbaum also reviewed on-going clinical trials looking at the efficacy of oxytocin on social and attention deficits (NCT02710084) and a phase I clinical trial of AMO-01 IV infusion for the treatment of epilepsy in adolescents with PMS (NCT03493607). AMO-01 is a Ras-ERK pathway inhibitor, developed after observation of ERK pathway overactivity in the Shank3 mice. The AMO-01 compound rescued the phenotype in the Shank3 mice, including seizure activity [10]. This clinical trial and others are reviewed in Table 3.

**Table 3:** Summary of clinical research that is on-going or completed for PMS, including both interventional and observational studies

Study title	Study design	Status
Interventional Studies		
Growth Hormone Treatment in Children with Phelan-McDermid Syndrome	Recombinant Human Growth Hormone	Recruiting
Piloting Treatment with Intranasal Oxytocin in Phelan-McDermid Syndrome	Oxytocin vs Placebo (Saline)	Recruiting
Clinical Trial in 22q13 Deletion Syndrome	Insulin-like Growth Factor-1 (IFG-1)	Recruiting
AMO-01 to Treat Adolescents and Adults with Phelan-McDermid Syndrome (PMS) and Co-morbid Epilepsy	AMO-01	Recruiting
Is there an effect of Intranasal Insulin on Development and Behavior in PMS?	Intranasal Insulin	Completed
Intranasal Insulin to Improve Developmental Delay in Children with 22q13 Deletion Syndrome: An Exploratory Clinical Trial	Intranasal Insulin	
Observational Studies		
Mitochondrial Dysfunction in Phelan-McDermid Syndrome: Explaining Clinical Variation and Providing a Path Towards Treatment	Observational	Completed
Mapping the Phenotype in Adults with Phelan-McDermid Syndrome	Observational	Recruiting
Mapping the Genotype, Phenotype, and Natural History of Phelan-McDermid Syndrome	Observational	Active, Not Recruiting

**Fig. 1:**
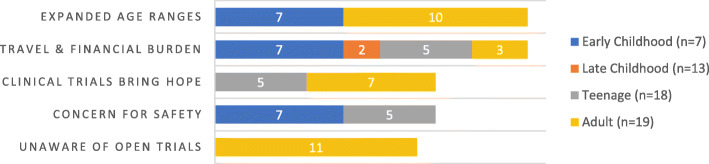
Clinical Trials Update table discussion topics of concern. Demonstrates the top five concerns raised by families during the clinical trials session. Families of both young children and adult patients expressed concerns that clinical trials included narrow age ranges, while all age groups expressed concerns regarding the travel and financial burden to families who participate in research studies

Table discussions revealed concerns across the following major themes: 1) desire for trials with inclusion of broader age ranges, 2) location and cost of trials, 3) concern for safety of trial participants, 4) a belief that trials are important and bring hope to families, and 5) lack of knowledge of active trials (See Fig. 1). The panel discussion touched on these themes. The panel empowered families to review active studies on www.clinicaltrials.gov as well as the lengthy drug-approval process in the US. Additionally, the panel reviewed the primary goals of the FDA to bring safe and efficacious drugs to patients, necessitating rigorous and well-designed studies to expedite that process.

### Session 2: Epilepsy, Jimmy Holder, MD, PhD, Baylor College of Medicine

This session gave an overview of seizure semiology, definitions, and work-up, as well as a review of published literature on epilepsy in PMS. A prevalence of 14 to 70% is reported in 12 retrospective studies of epilepsy in PMS, and 41% in one prospective study [11]. One study suggests that risk of epilepsy may increase with age with a prevalence of 60% in patients of 18-years old compared to 30% of all reviewed patients in one cohort [12]. In a case series of six Italian patients with 22q13.3 deletion syndrome, 3/6 had epilepsy (though one with a history of meningitis), 1/6 carried a deletion that did not involve *SHANK3*, and all 6 had a “benign course” as described by the authors [13]. Holder et al analyzed the clinical data of 24 patients with PMS in their practice and report a prevalence of at least one lifetime seizure in 46% (11 of 24) with a mean age of onset of 5.2 years (range 14 months to 14 years). The seizure burden ranged from 1 to 100 per day and the most common seizure type was atypical absence, though generalized tonic-clonic, atonic, myoclonic, and focal seizures were also reported. Status epilepticus was reported in 20% of patients and 18% also met criteria for Lennox-Gastaut Syndrome. EEGs were abnormal in 67% of patients: 1) slowing or absence of an occipital dominant rhythm (42%), 2) focal spike and wave discharges (38%), and 3) generalized spike and wave discharges (19%). There was no correlation between brain MRI abnormalities and abnormal electrical activity on the EEG [11]. Preliminary data from functional MRI studies in PMS reveal sparse association networks compared to normal controls, but studies are still on-going. No specific anti-seizure medication was found to be superior. Finally, Dr. Holder reviewed unpublished preliminary data generated by the on-going PMS natural history study which has performed longitudinal analysis of EEG in 16 patients with PMS and found 43% have epilepsy. Generalized slowing is the most frequently seen abnormality. Interestingly, the prevalence of epileptiform discharges on EEG increased to 75% when EEG recordings were extended overnight compared to 25% of routine EEGs, but no seizures were captured on any of these EEG recordings (*unpublished data*). Finally, there is some data to suggest that patients who carry a point mutation of *SHANK3* may be more prone to seizures [14].

**Fig. 2:**
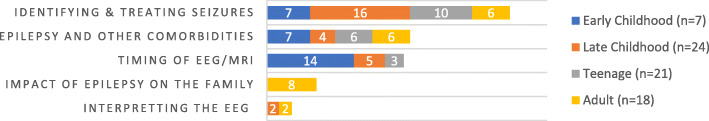
Epilepsy table discussion topics of concern. Demonstrates the top seven concerns raised by families regarding epilepsy. Across all age groups, families worried about the ability to accurately identify and treat seizures. Additionally, many families raised concerns about how the presence of epilepsy may impact other aspects of the PMS patient’s health such as developmental regression

Table discussions revealed concerns across the following major themes: 1) concern about identifying and treating seizures, 2) concern about the timing and indication for EEG and MRI, 3) confusion over the interpretation of EEG results, 4) burden of recording and logging seizures accurately, 5) an interest in the relationship between seizures and developmental regression, puberty, and age of onset, and finally, 6) worry over how epilepsy might impact their family (See Fig. 2). The panel discussion touched on these themes. The panel reported a lack of evidence of any correlation between age of onset of seizures and pubertal changes. There is no clear age at which PMS patients are at a higher or lower risk of developing seizures. Further, the role of EEG results in the care of patients with neurodevelopmental disorders is still under investigation in many disorders, in addition to PMS. For now, the EEG must be interpreted in the context of the individual patient’s presenting symptoms and can actually normalize with time. Further research in this area is needed. The panel also addressed questions regarding the role of cannabidiol in PMS and agreed it would be a worthy future investigation, though cautioned that it is important to monitor levels of concomitant medications closely.

### Session 3: Challenging Behaviors, Nathan Call, MD, Emory University School of Medicine

This session provided an overview of challenging behaviors that are encountered by many families affected by neurodevelopmental disabilities in general. Dr. Call acknowledged the pervasiveness of behavioral disorders across many disorders, but noted the exact prevalence is difficult to ascertain secondary to the subjectivity of the reporting. Notably, behavior problems can have a negative impact on both the individual and the caregivers. For the individual, aggression and irritability can lead to physical harm and stigmatization and exclusion from the community [15, 16]. For the caregivers, these challenging behaviors negatively impact family functioning, diminish marital satisfaction, lead to higher rates of behavior problems in siblings [17, 18], and ultimately may lead to parental unemployment [16, 19, 20]. Dr. Call also reviewed the two foundational approaches to intervention: psychopharmacology and behavioral therapy. Iwata et al. [21] sought to understand the function of the behavior and developed a model of “Function-based treatment.” In this model, the treatment team seeks to understand the purpose the behavior is serving for the individual and divides behaviors into two broad categories: 1) social behavior which requires another person to meet the goal (i.e. needing mom to get me a cookie), or 2) an automatic behavior which does not require anyone else to meet the goal (i.e. hitting self in the head because it feels good). Over 40 years of research and more than 170 publications across over 25 journals have yielded inconclusive results with limited generalizability secondary to small sample sizes and a bias to publish only positive results. Finally, Dr. Call reviewed several areas of on-going research in the treatment of pica, encopresis, and development of wearable devices designed to detect aggression in a quantifiable manner. For pica, Call et al. employed a function-based treatment model and demonstrated improvement in pica in 13 children with developmental disabilities who were seen at the Severe Behavior Program at Marcus Autism Center [22]. His group has also demonstrated significant improvement in encopresis by using a multidisciplinary approach after 4 weeks of intensive therapy in a small group of 20 patients [23]. They are planning to expand to a larger cohort of 150 patients with autism spectrum disorder. Finally, his group is working with biotechnology engineers to design a wearable device that will discern between aggressive and non-aggressive movements. Preliminary results showed an overall accuracy of 80.3%, specificity of 41.5%, and sensitivity of 95.4%. This study is on-going (NIH R21 MH104363).

**Fig. 3:**
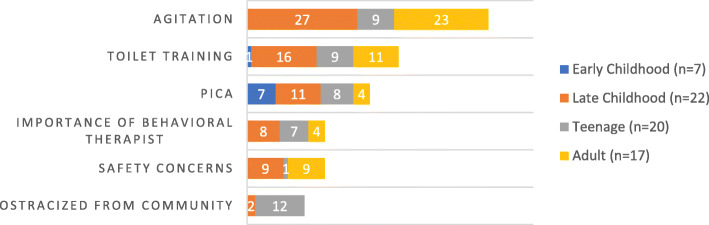
Challenging Behaviors table discussion topics of concern. Demonstrates the concerns raised by families during the challenging behaviors session. Overall, families of young children had fewer concerns regarding this topic, while agitation was seen throughout all older age groups. Additionally, struggles with toilet-training were commonly reported among families of older patients

**Fig. 4:**
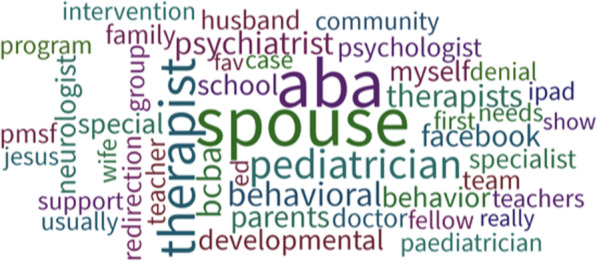
PollEverywhere Infographic: Who do you turn to when your child has problem behaviors? Is an infographic generated from a poll of PMS families reflecting on who they turn to when their child has challenging behaviors. The font size positively correlates with frequency of reporting by parents. The most frequently reported responses were the spouse, applied behavioral analysis (ABA) therapist, and pediatrician

Table discussions revealed concerns across the following major themes: 1) general concerns about aggression and agitation ranging from hitting/biting to screaming/fits of laughter, 2) concern about toilet-training, 3) managing pica, 4) the importance of working with a behavioral therapist on new strategies and identification of triggers, 5) worry about their child’s safety, and finally, 6) feeling ostracized from the community (see Fig. 3). Additionally, PollEverywhere data revealed families/caregivers most often turn to their spouse and behavioral therapists for support when they are struggling with challenging behaviors (see Fig. 4). The panel discussion touched on these themes, primarily focusing on toilet-training, analysis of behavioral triggers, and management of pica. Dr. Call reviewed the approach the Marcus Autism Center takes including a 2-week inpatient toileting program based on positive reinforcement of successful attempts at toileting. They also work closely with GI colleagues to address an underlying physiological reason for incontinence. The panel also emphasized the pervasiveness of this problem across many developmental disorders including Fragile X Syndrome in which 20% of patients don’t attain independent toileting by 18-years of age, and this was found to correlate with their ability to communicate. More structured toileting regimens may be needed in non-verbal patients. Dr. Call also reviewed the Marcus Autism Center’s approach to pica, involving teaching children with disabilities to exchange non-food items for valued items, such as a favorite snack. Finally, the panel reinforced the importance of carefully analyzing behaviors which seem unprovoked as many of these seemingly random acts are still an attempt to communicate a need.

### Session 4: Genetics 101, Pilar Magoulas, MS, GC, Texas Children’s hospital

In this session, Pilar Magoulas reviewed basic genetic concepts including chromosomal organization terminology, types of genetic variants, and methodologies available for genetic testing. She also reviewed the genetics of PMS, specifically. The majority (80%) of PMS individuals carry a deletion involving the long arm of chromosome 22 (22q13.3), and the remaining 20% are ring formations or translocations. The average deletion size is 4.5 Mb. There are up to 108 genes in this region, and larger deletions tend to involve more genes. It is well-accepted that the minimal critical region to manifest symptoms of PMS involves the *SHANK3* gene. It is controversial whether deletions which spare SHANK3 should be classified as PMS. *SHANK3* encodes a protein which is important for neuronal synaptic development. Loss of one functional copy of *SHANK3* is sufficient to cause neurologic dysfunction that will manifest as learning difficulties, intellectual disability, language delay, and behavioral problems.

**Fig. 5:**
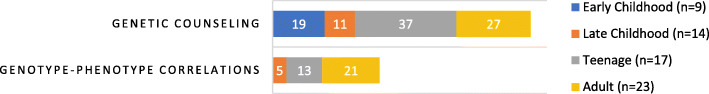
Genetics 101 table discussion topics of concern. Demonstrates the top two concerns raised by families during the Genetics 101 session. All age groups expressed the importance of working with a genetic counselor to fully understand the details of the genetic report and estimated risk to other family members. Additionally, older age groups were interested in understanding the relationship between clinical presentations and molecular genotypes, including the role of genes other than SHANK3

Table discussions revealed concerns across two major themes: 1) importance of genetic counseling to understand their genetic reports and estimated risk to other family members and 2) genotype-phenotype correlations (Fig. 5). The primary concern among the parents was when parental testing was indicated. Most families agreed working with genetic counselors was valuable to help interpret genetic reports and to guide indications for testing of additional family members, including parental testing. Many families are also interested in the role of other genes in the region, although the expert panel explained that the vast majority of PMS-related symptoms can be explained by *SHANK3* dysfunction. The panel also stressed the importance of participating in the PMS International Registry to further explore genotype-phenotype correlations, which is being used to generate hypotheses to systematically study in addition to serving as a referral source for future clinical trials.. Finally, families discussed the types of genetic testing available with a focus on options for prenatal testing. The expert panel reviewed currently available tests including amniocentesis or chorionic villus sampling, which can test for known familial mutations. Non-invasive prenatal testing (NIPT), a maternal blood test to detect fragments of fetal DNA, however, is only available for common trisomies and select microdeletions of at least 7 Mb in length.

### Session 5: Gastrointestinal Dysfunction, Kent Williams, MD, Nationwide Children’s, gastroenterologist

In this session, Dr. Williams provided an overview of common gastrointestinal complications associated with neurodevelopmental disorders, especially constipation, which affects over half of the patients in his GI autism spectrum disorders clinic. He stressed the importance of recognizing the signs and symptoms of constipation: hard consistency, excessively large stool, effortful or painful defecation, stool incontinence, and decreased frequency of bowel movements. He emphasized that untreated constipation can lead to megarectum in extreme cases and can be associated with worsening behavior problems. Dr. Williams reviewed common interventions used to manage chronic constipation including laxatives, stimulants, and ultimately procedures such as antegrade enema therapy and Botox injections of the anal sphincter. Finally, Dr. Williams addressed the concerns about associations between Miralax and neuropsychiatric symptoms such as seizures, tremors, obsessive-compulsive behaviors, and mood lability. In a Nationwide Hospital study, researchers demonstrated that administration of PEG 3350 (active compound in Miralax), was not associated with sustained elevation of glycol levels. They analyzed blood samples from pediatric patients exposed to PEG 3350 (*n* = 9) and compared those to an unexposed control group (*n* = 18). All participants in the study had detectable levels of ethylene glycol and di-ethylene glycol. Importantly, they report that a 10-kg child would have to consume 1 l of water mixed with 50 capfuls of Miralax in a single day in order to reach toxic exposure levels of ethylene glycol [24]. Dr. Williams concluded his session by reviewing preliminary data that suggests patients with autism have a unique microbiome profile, an area of research ripe for biomarker discovery.

**Fig. 6:**
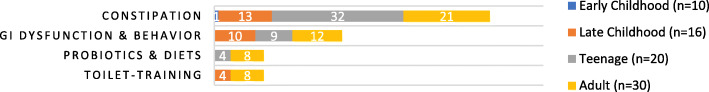
Gastrointestinal Dysfunction table discussion topics of concern. Demonstrates concerns raised by families during the Gastrointestinal Dysfunction session. Diagnosis and management of constipation was the major concern raised by the families, followed by the relationship between worsening constipation and challenging behaviors

Table discussions revealed concerns across four major themes: 1) diagnosis and management of constipation 2) relationship between constipation and behavior, 3) use of probiotics and specialized diets, and 4) toilet-training (Fig. 6). Most families agreed that constipation was a significant problem that affected their daily lives and that behavior tended to worsen when constipation was under poor control. As discussed in other sessions, struggles with toilet-training were again addressed. During the table discussions, families reviewed various supplements and special diets utilized, however during the expert panel portion of the session families were cautioned of the lack of FDA regulation of the probiotic industry and importance of working with a registered dietician when implementing a restrictive diet. Further, the panel urged families to be thoughtful in analyzing targeted behavioral outcomes over a specific time interval in order to assess the efficacy of these interventions, which are generally low risk but can cause financial stress and burdens to the family. Because there was such a great interest in the GI dysfunction session, researchers agreed to meet with small groups of families, grouped by their child’s age to learn more about the challenges they face.

### Session 6: Developmental Regression, Jon Bernstein, MD, PhD, Stanford University School of Medicine

In this session, Dr. Bernstein reviewed the published literature on developmental regression in PMS. As defined by the NIH Genetic and Rare Disease Information Center, “developmental regression refers to a condition in which children… start to lose the skills they developed and the developmental milestones they have met” [25]. Developmental regression is widely reported in the PMS literature, however the typical age of onset and correlations between comorbid neurological conditions remains elusive. Across 13 publications that discuss regression or psychiatric comorbidities in PMS, the onset of regression is highly variable and inconsistently evaluated in relation to medical comorbidities and genotype [2, 14, 26–30]. Dr. Bernstein raised the hypothesis that developmental regression may have a unique profile in PMS and further research is needed to elucidate the relationship between regression and medical comorbidities and genotype as well as potential interventions.

**Fig. 7:**
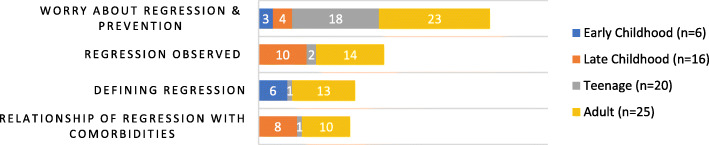
Developmental Regression table discussion topics of concern. Demonstrates the concerns raised by families during the Developmental Regression session. The emergence of developmental regression was raised by many families, especially with regards to way in which regression might be prevented

Table discussions revealed concerns across five major themes: 1) worry about regression and desire for mechanisms of prevention, 2) the prevalence of regression among attendees, 3) how to define regression, and 4) association between regression and medical conditions (Fig. 7). Developmental regression was primarily reported by families with older children (0/6 Early Childhood, 3/6 Late Childhood, 3/20 Teenage, 13/19 Adults), and none of the skills that were reportedly lost were ever regained. There was great confusion around how to classify and identify regression and most agreed it was easier to identify regression in individuals who had attained higher levels of neurocognitive functioning. Further, the panel admitted the field currently lacks the ability to prevent regression at this time but suggested that maintaining adequate health and early identification may improve outcomes. Finally, the panel agreed that further investigation into the correlation between regression and genotypes are needed to understand this relationship and implement the appropriate surveillance.

A post-conference survey was sent electronically to all conference attendees. There were 85 respondents, representing approximately 50% of attendees. Of the 85 respondents, 90% participated in the McPosium, 96% strongly agreed or agree that they were satisfied with their experience overall, and 80% of respondents were very satisfied or satisfied with the format of the McPosium. In comments, some respondents highlighted the value of having a researcher and/or doctor at each table. The top three McPosium topics rated as “Critically Important Issues” included GI Dysfunction (65%), Regression (46%), and Genetics (37%).

## Discussion

The Phelan McDermid Syndrome Foundation (PMSF) was founded in 2002 to meet the needs of a growing community of Phelan McDermid Syndrome patients and families. The mission of the PMSF is “to improve the quality of life of people affected by PMS worldwide by providing family support, accelerating research and raising awareness.” The McPosium was born out of necessity in 2016 and has grown into an endeavor that puts families, clinicians, and researcher at the same table to align goals for this rare and vulnerable population. By hosting sessions on core features important to the community, the scientists are given the opportunity to plan future projects on patient-centered concerns and build collaborations across disciplines. This format also empowers families to voice their concerns in a less intimidating forum and gain more understanding of how to navigate the complex world of basic and clinical research. Past conferences have changed the landscape of clinical and translational research on PMS. In response to concerns raised by families at the 2016 McPosium, the PMSF funded an adult natural history study to further characterize the progression of disease beyond childhood. Development of a Shank3 zebrafish modeling the GI symptoms of PMS was established by Dallman et al. after attending the 2016 McPosium and learning about the burden of GI dysfunction on the PMS community [31]. Given the high rate of autism spectrum in PMS, Dallman’s group is hopeful that knowledge gained from this work will also inform the autism community at large. Further, concentration on the burden of GI symptoms on this community spurred additional studies of the microbiome by Dr. Ruth Ann Luna’s group, who collected stool samples from the 2018 conference attendees.

Among the attendees to the 2018 McPosium, families were highly engaged with the McPosium sessions and provided insights into how each topic affected their daily lives. In general, the Early Childhood parents raised the fewest number of concerns. This may be explained as many of the manifestations of the syndrome seem to emerge with time and often the struggles of the infant and toddler years overlap with struggles of typically-developing children. Families with PMS patients of all ages expressed concerns surrounding participation in Clinical Trials. Concerns regarding epilepsy and behavioral problems were raised more by late-childhood families, while teen and adult families raised more concerns regarding genetics, GI dysfunction, and regression. This pattern of age distribution across the various sessions may correlate with the natural history of the syndrome as different symptoms are likely to become more prominent at differing ages.

Upon completion of the 2018 PMSF International Family Conference and McPosium, the PMSF Operations Plan for 2020 was initiated to address all three program areas including research, family support, and advocacy/awareness. Each program includes a measurable outcome such that final conference reports, family needs assessments, and the patient voice are embedded in the planning of subsequent events. The theme of the 2020 PMSF International Family Conference is “Welcome Home” after overwhelming feedback from families that they felt supported through their interactions with other families and the community of researchers in 2018. The 2020 conference is projected to broaden the scope of the research track. Members of the newly formed Medical and Scientific Advisory Committees are invited, in addition to several international researchers to present on their work on PMS and SHANK3. Additionally, based on feedback from the 2018 McPosium, there will be a major focus on how specific genetic variants impact PMS patients and families. This will include individual informational sessions, panel discussions, and ample time for families and investigators to network and share ideas and experiences.

## Conclusion

Collectively, rare diseases are actually more common than AIDs and cancer combined, affecting more than 350 million people worldwide, and over 95% of these disease lack an FDA-approved treatment [32]. Disease-specific foundations give patients and families a platform and community of support through which they can learn from one another’s experience and organize a collective voice to advance research based on their needs and interests. The PMS Foundation continues to refine their approach to steering research efforts and community support through the McPosium at their annual international family conference. This meeting format provides a unique opportunity for clinicians and investigators to interact with numerous families in one setting and even collect biological samples or clinical data from eligible participants. Traveling is a common barrier to participating in research for families with children with developmental disabilities. In this format, the families are able to travel to a single site and accomplish numerous tasks including socializing with families living with similar struggles, receive updates directly from researchers and clinicians, and voice their current concerns and shape future directions for the community. The challenges that the PMS foundation has faced are not unique to their patient population as many rare neurodevelopmental disorders disproportionately affect children and are often associated with multiple medical comorbidities including behavioral challenges and mobility problems. The McPosium model has been successful in generating thoughtful research questions that lead to innovative investigations and finally clinical trials of potential therapies. Though organization of events of this scale are cumbersome and expensive, the investment in the disease community, both families and scientists, is invaluable tool that could be employed by any rare disease group.

## Methods

A family needs/impact survey was issued to the PMS community to confirm the topics for the 2018 McPosium prior to the event. This was cross-referenced with data from the PMSF International Registry. Based upon these results, the 2018 Phelan-McPosium included six topics of high interest to the PMS community: 1) Clinical Trials, 2) Epilepsy, 3) Challenging Behaviors, 4) Genetics 101, 5) GI Dysfunction, and 6) Developmental Regression. Each topic included an introductory 20-min lecture by an expert in the field, followed by a 20-min family roundtable discussion with researchers, and concluded with a 20-min panel discussion about the topic and future directions. A PMS family member was designated as the table leader and note taker and was accompanied by at least one representative PMS researcher was present at each roundtable. PMSF partners with both domestic and international groups to facilitate attendance to the conference as well as to disseminate information generated by the foundation. As travel can be challenging with an affected person and can pose a financial burden to families, PMSF is committed to breaking down barriers to attendance to the International Conference. Scholarships are offered for first-time attendees, and the registration fee can be waived for any family based on financial need. PMSF also livestreams the conference and records the McPosium sessions to extend the dissemination of information globally. PMSF uses social media channels and a conference-specific mobile app to help families who are not able to attend to participate in sessions. The entire conference is also streamed to a designated on-site “quiet room” so that families may continue to participate in the meeting when affected PMS family members are overstimulated in the main conference room.

Families and lay-public were the target audience for the introductory lectures in the Phelan-McPosium. Each lecture was given by a subject matter expert and covered the current state of knowledge for each topic in PMS or other related neurodevelopmental disorders such as autism and intellectual disability. Following each lecture, table participants discussed concerns with regards to the topic. Families were organized by age of the PMS-individuals within the conference room, and for ease of data analysis, the groups have been condensed to Early Childhood (< 6 years old), Late Childhood (7 to 12 years), Teenage (13 to 19 years), Adult (> 20 years). A representative PMS family member from each age-group classification was trained to be a table facilitator and took notes during the roundtable discussions, recording table attendees, detailed description of concerns, rating of concern (Likert scale of 1 to 5, 1 = very low concern, 5 = very high concern), and number of parents actively participating in the discussion. Table facilitators were volunteer family members who underwent a 60-min training the day prior to the conference. They were provided with note-taking templates and sample questions for discussion. These notes were reviewed by the authors and organized systematically by theme, age, and level of concern to the families in a database. Parents also shared opinions and questions with the group at large through the PollEverywhere app. Questions and concerns posted to the PollEverywhere app were displayed during the roundtable and panel discussions. Through the PollEverywhere app, attendees were able to up-vote or down-vote questions, generating a trending score. The panelists then were asked to discuss how current and future research would address the priorities of families affected by PMS in addition to questions ranked by parents and caregivers through the PollEverywhere app. Questions receiving a trending score of 25 or greater are reported within each topic. A post-conference survey was issued to all attendees. Responses are being used to plan the 2020 PMSF International Conference.

## Data Availability

The datasets used and/or analyzed during the current study are available from the corresponding author on reasonable request.

## Abbreviations

PMS: Phelan-McDermid Syndrome
PMSF: Phelan-McDermid Syndrome Foundation
IGF-1: Insulin-like growth factor
EEG: Electroencephalogram
MRI: Magnetic Resonance Imaging
NIPT: Non-invasive prenatal testing
